# Ecofriendly production of silver nanoparticles using *Candida utilis* and its mechanistic action against pathogenic microorganisms

**DOI:** 10.1007/s13205-014-0196-y

**Published:** 2014-01-24

**Authors:** Shailesh R. Waghmare, Mustopa N. Mulla, Suryakant R. Marathe, Kailas D. Sonawane

**Affiliations:** 1 Department of Microbiology, Shivaji University, Kolhapur, 416004 Maharashtra India; 2 Department of Biochemistry, Shivaji University, Kolhapur, Maharashtra India

**Keywords:** Ecofriendly, Silver nanoparticles, *Candida utilis*, Antibacterial, *Pseudomonas aeruginosa*

## Abstract

Silver nanoparticles (AgNPs) have attracted great interest due to their applications in various areas. In the present study ecofriendly biosynthesis of extracellular silver nanoparticles was carried out using *Candida utilis* NCIM 3469. Characterization of synthesized AgNPs was done by UV–visible spectroscopy, Scanning electron microscopy and antibacterial activity. AgNPs are found spherical in shape with size in the range of 20–80 nm. AgNPs showed antibacterial activity against pathogenic organisms such as *Pseudomonas aeruginosa, Staphylococcus aureus,* and *Escherichia coli*. The SEM analysis confirms the antibacterial activity of Ag nanoparticles due to damage of cytoplasmic membrane. AgNPs synthesized by *C. utilis* could be applicable in the development of antibacterial water filters for treatment of water.

## Introduction

Silver nanoparticles have recently attracted a lot of interest due to their distinctive properties such as large surface areas, unique physical, chemical and biological properties (Sharma et al. 2009; Zhou et al. 2009). Silver nanoparticles are emerging as a new generation of antibacterial agent (Rai et al. 2009) medical applications (Atyeh et al. 2007; Chang and Weinstein 1975), and antibacterial water filter (Jain and Pradeep 2005). The outbreak of infectious diseases caused by different pathogenic bacteria as well as the development of antibiotic resistance (Hsueh 2010; Kumarasamy et al. 2010; Pitout 2010) has prompted pharmaceutical companies and researchers to search for new antibacterial agents. Among nanomaterials like copper, zinc, titanium, magnesium, silver, gold, and alginate, silver nanoparticles have proved to be the most effective as they have good antibacterial efficacy against bacteria, viruses and other eukaryotic microorganisms (Rai et al. 2009). Different studies have been performed to check the bactericidal effect of silver nanoparticles against Gram negative and Gram positive bacteria, but the bactericidal mechanism of this compound has not been clearly elucidated. Morones et al. (2005) studied the antibacterial activity of silver nanoparticles against four types of Gram negative bacteria, *Escherichia coli*, *V. cholera, Pseudomonas aeruginosa* and *S. typhus*. They have observed that these silver nanoparticles are attached to the surface of the cell membrane and disturb the function of cell membrane, penetrate bacteria, and release silver ions.

Water is the common breeding ground for many pathogens. In countries such as India, 80 % of the diseases are due to bacterial contamination in drinking water. The removal or inactivation of pathogenic microorganisms is the last step in the treatment of wastewater. During the past few decades, several investigations have been carried out concerning the use of metal ions (Ag, Cu, Zn, Hg, Ti, Ni, Co) as bactericides for water disinfection (Feng et al. 2000; Islam et al. 2003). The use of metal nanoparticles for water disinfection is relatively new (Stoimenov et al. 2002; Zhang et al. 2003). Because of their high reactivity due to the large surface to volume ratio (Ichinose 1992), nanoparticles are expected to play a crucial role in water purification (Stoimenov et al. 2002; Zhang et al. 2003) when water becomes an important commodity (Barraque, 2003). Pesticide removal from drinking water with the help of nanoparticles was reported by Nair and Pradeep (2004).

Although, various chemical and biochemical methods are being explored for the AgNPs production, but microbes are exceedingly effective in this process (Narayanan and Sakthivel 2010). Biosynthesis of silver nanoparticles from bacteria, fungi, yeast, plants, and fruits have been reported (Kowshik et al. 2003). Based on their enormous biotechnological applications, microorganisms such as bacteria, fungi, and yeast are now regarded as possible ecofriendly “nano-factories” (Ahmad et al. 2002). The detail mechanism of microbial synthesis of Ag nanoparticles is yet to be elucidated, but several reports suggested role of certain enzymes such as NADH-dependent reductase from *Fusarium oxysporum* (Ahmad et al. 2002), proteins from *Phanerochaete chrysosporium* (Vigneshwaran et al. 2006) and *Plectonema boryanum* (Lengke et al. 2007) in the synthesis of nanoparticles. Here, we first time report the biofabrication of silver nanoparticles using *Candida utilis* and its application as antimicrobial agent against pathogenic microorganisms.

## Materials and methods

### Microorganisms

The cultures of *C. utilis* NCIM 3469, *E. coli* NCIM 2832, *Salmonella typhimurium* NCIM 2501, *Candida albicans* NCIM 3466 and *P. aeruginosa* NCIM 5032 used in this study were obtained from National Collection of Industrial Microorganisms (NCIM), Pune, Maharashtra, India.

### Biosynthesis of silver nanoparticles

The *C. utilis* was grown on MGYP medium containing malt extract 3.0, glucose 10.0, yeast extract 3.0, peptone 5.0 and agar 20.0 in 1,000 ml distilled water. The fresh culture of *C. utilis* was inoculated in 100 ml mineral medium containing NaNO_3_: 3, K_2_HPO_4_: 1, KCl: 0.5, MgSO_4_·7H_2_O: 0.5, FeSO_4_: 0.01, yeast extract 0.1, peptone: 0.5 per 1,000 ml distilled water and flask was incubated at 30 °C for 48 h. After incubation the cells were separated by centrifugation at 5,000 rpm for 15 min and 100 ml supernatant was collected in 250 ml Erlenmeyer flask containing 10 ml AgNO_3_ solution (contain 37.18 mg AgNO_3_) which makes the final concentration of AgNO_3_ 2 mM. The flask was then incubated at 30 °C on shaking incubator at 120 rpm in dark. After 6 h, the sample was taken out for UV–visible (UV–Vis) spectrophotometric analysis. Another flask containing a mixture of 100 ml supernatant and 10 ml AgNO_3_ solution (contain 37.18 mg AgNO_3_) was autoclaved at 120 °C for 15 min and further analyzed by UV–Vis spectrophotometer.

In a separate experiment, culture of *C. utilis* was grown in 100 ml mineral medium as described above for 24 h. After incubation, cell biomass was collected and then added in 2 mM 100 ml AgNO_3_ solution and incubated at shaking condition. The AgNO_3_ reduced to AgNPs shows change in color of AgNO_3_ from colorless to brown was monitored after 24 h for production of AgNPs by spectrophotometric analysis between the wavelength ranges 300 to 700 nm. The effect of different concentrations of AgNO_3_ such as 2, 4, 6, 8, and 10 mM on synthesis of AgNPs was monitored for 24 h.

### Characterization of AgNPs

The formation of AgNPs was monitored by UV–Vis spectroscopy by recording the spectra between 300 and 700 nm and simultaneously monitoring the appearance of the characteristic peak at 39–420 nm using Shimadzu U-1800 double beam spectrophotometer. The suspension obtained at the time point of maximum production of silver nanoparticles was air dried and then subjected to SEM, using JEOL JSM 6360 (Japan) Scanning Electron Microscope and micrographs were taken.

### Antibacterial activity of AgNPs

AgNPs synthesized using whole cells of *C. utilis,* supernatant with heat treatment and 6 mM aqueous solution of silver nitrate as control were tested for antibacterial activity against pathogenic microorganisms such as *S. aureus, P. aeruginosa, and E. coli*. The antibacterial activity was tested by the agar disc diffusion method (Kim et al. 2007) and zone of inhibition was noted.

To examine bactericidal effect on Gram negative organism, *P. aeruginosa* was used as model organism. The fresh culture of *P. aeruginosa* was inoculated in the nutrient broth and then incubated at 37 °C for 24 h. 100 μl of nanoparticle solution having concentration 1 mg ml^−1^ was added in the nutrient broth containing *P. aeruginosa*. After 2 h of incubation, the sample was prepared according to method described by Galabova et al. (1996) for SEM analysis.

### Statistical analysis

Results obtained were the mean of three or more determinants. Analysis of variance was carried out on all data at *p* < 0.05 using Graph Pad software (GraphPad InStat version 3.00, GraphPad Software, San Diego, CA, USA).

## Results

### Biosynthesis of Ag nanoparticles

**Fig. 1:**
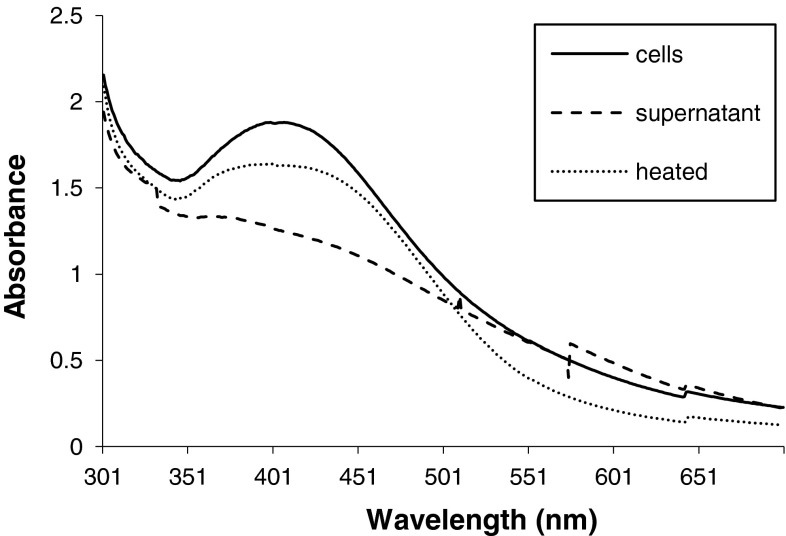
UV–Vis spectroscopy of synthesized silver nanoparticles. Synthesis of silver nanoparticles was carried out using cells of *C. utilis*, supernatant and supernatant with heat treatment process

The aqueous AgNO_3_ was reduced to metallic silver. The synthesis of silver nanoparticles was monitored in three flasks: (1) in presence of supernatant at 30 °C; (2) in presence of supernatant at temperature 120 °C for 15 min; (3) in the presence of cell biomass of *C. utilis*. It has been observed that the silver nanoparticles were produced when AgNO_3_ mixed with supernatant and heated at 120 °C for 15 min, whereas no brown color was seen in the flask which was kept at 30 °C. These results indicate the role of temperature in the synthesis of silver nanoparticles. In the case of flask containing cell biomass of *C. utilis* with AgNO_3_, it was found that the intensity of brown color was increased as the incubation time increases at 30 °C. It confirms the synthesis of silver nanoparticles by *C. utilis*, as shown in Fig. 1.

### Characterization of AgNPs

**Fig. 2:**
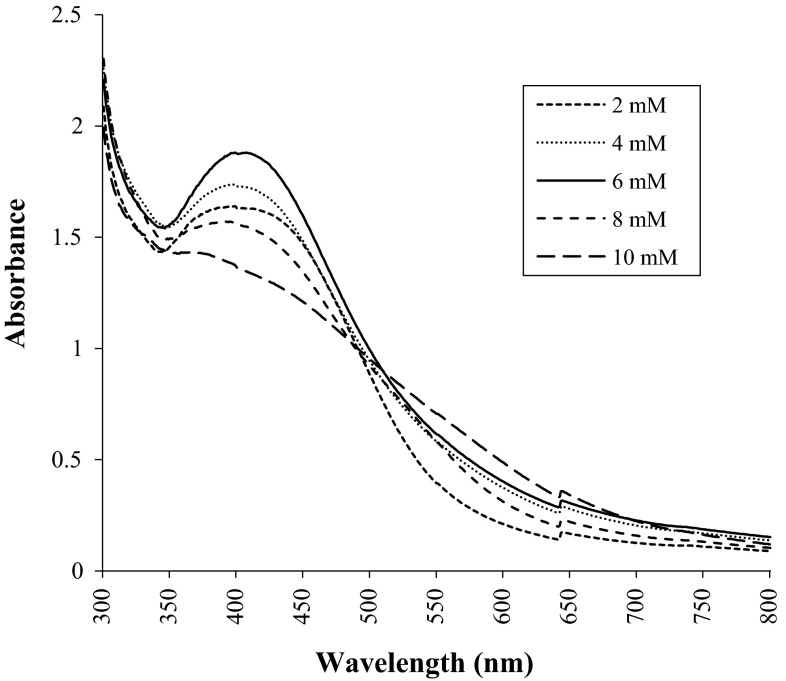
Effect of silver nitrate concentration on synthesis of AgNPs. Synthesis of AgNPs was carried out at various concentration of silver nitrate such as 2, 4, 6, 8, and 10 mM using cells of *C. utilis*

**Fig. 3:**
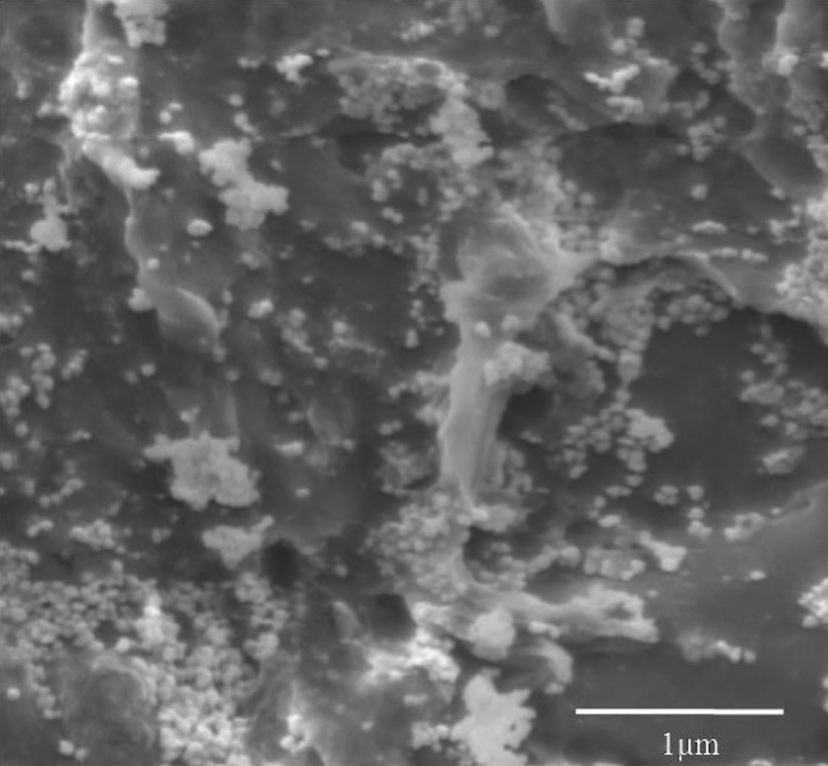
Scanning Electron Microscopy of AgNPs. AgNPs synthesized by *C. utilis* analyzed for morphology by SEM

After 24 h of incubation the aqueous silver nitrate turned colorless to brownish yellow in the presence of *C. utilis* cell biomass. UV–Vis spectra of solution revealed peak between 401 and 410 nm, with maximum absorbance at 406 nm. In the present study we have studied the effect of AgNO_3_ concentration on the synthesis of silver nanoparticles. As the concentration of AgNO_3_ increases from 2 to 6 mM, synthesis of AgNPs increases, whereas by increasing the concentration of AgNO_3_ up to 8 mM the synthesis of AgNPs sharply decreases as depicted in Fig. 2. The UV–Vis spectra of aqueous silver nitrate solution when monitored separately did not show any peak between 40 and 410 nm. SEM micrograph of synthesized silver nanoparticles revealed the formation of spherical nanoparticles with the size range of 20–100 nm along with some nanoclusters (Fig. 3).

### Antibacterial activity of synthesized AgNPs

**Fig. 4:**
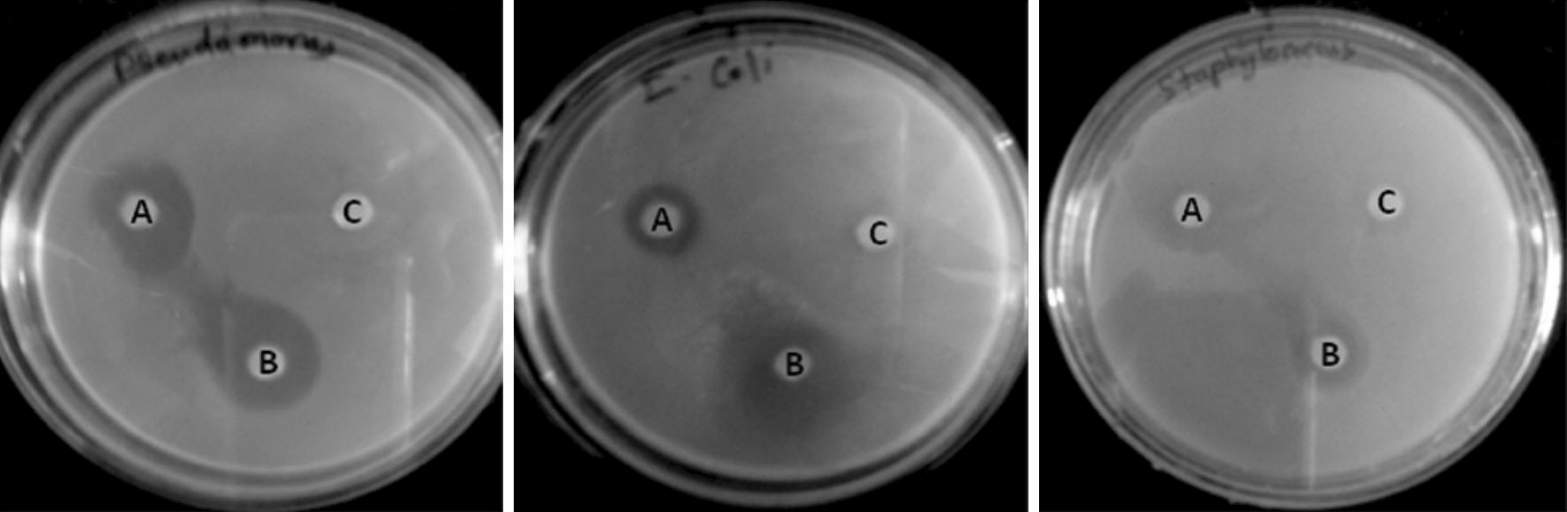
Antibacterial activity of AgNPs against pathogens. Antibacterial activity of AgNPs synthesized by cells of *C. utilis* (**b**), Supernatant with heat treatment process (**a**) and control as aqueous solution of silver nitrate (**c**) were tested against pathogens such as *P*. aeruginosa, *E. coli*, and *S. aureus* by disc diffusion method

**Fig. 5:**
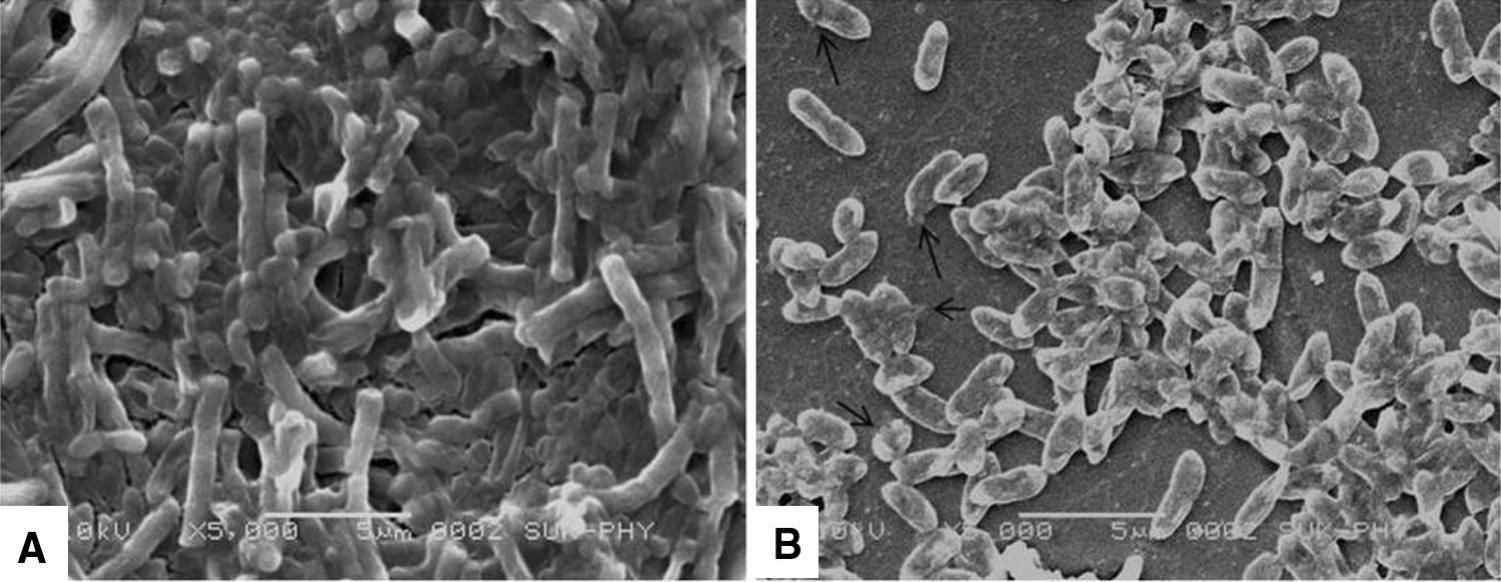
SEM analysis of *P.* aeruginosa after the treatment with synthesized Ag nanoparticles (**b)** and native cells (**a)**

The antibacterial activity of synthesized AgNPs by cell biomass of *C. utilis* (*B*), by the supernatant with heat treatment (*A*) and control as aqueous solution of silver nitrate (*C*) was tested on the Gram positive (*S. aureus*) and Gram negative (*P. aeruginosa*, *E. coli*) organism. AgNPs showed more antibacterial activity against Gram negative organisms such as *P. aeruginosa*, and *E. coli* as compared to Gram positive organism, i.e., *S. aureus* (Fig. 4). However, AgNPs synthesized using cell biomass showed more antimicrobial activity than the supernatant (Table 1). SEM was used to evaluate the surface morphology of the both native (Fig. 5a) and treated (Fig. 5b) *P. aeruginosa*. The SEM micrograph revealed the damage of cell wall after the treatment of AgNPs (Fig. 5b) by releasing cytoplasmic content of cells of *P. aeruginosa*.

**Table 1:** Antibacterial activity of AgNPs against pathogens

Test organisms	Zone of inhibition in diameter (mm)	
	A	B
*P. aeruginosa*	13 ± 1.2	20 ± 1.1
*E. coli*	10 ± 1.0	18 ± 1.0
*S. aureus*	8 ± 0.8	12 ± 0.9

Each value represents the mean ± standard error values

*A* AgNPs synthesized heat treatment process with supernatant

*B* AgNPs synthesized using live *C.* utilis cells

## Discussion

In this study, we have for the first time reported the extracellular synthesis of silver nanoparticles using *C. utilis*. The extracellular synthesis of silver nanoparticles had been reported earlier from other yeast such as salt-tolerant yeast MKY3 (Kowshik et al. 2003), *C. guilliermondii* (Mishra et al. 2011), *Geotrichum* sp. (Jebali et al. 2011). The extracellular synthesis of AgNPs would be of great advantage to industry, since it would minimize the steps involved in the purification of AgNPs. The color of the reaction solution turned from colorless to brown indicating the formation of silver nanoparticles similar to that observed in earlier study. This color arises due to the excitation of surface plasmon vibrations in the metal nanoparticles (Narayanan and Sakthivel 2010).

The silver nanoparticles were synthesized using cells of *C. utilis*, which are spherical nanoparticles with the size range of 20–80 nm along with some nanoclusters (Fig. 3), whereas salt-tolerant yeast MKY3 synthesizes hexagonal silver nanoparticles with the size range of 2–5 nm (Kowshik et al. 2003). Antibacterial activity of AgNPs synthesized by *C. utilis* was found to be inhibitory to both Gram positive as well as Gram negative groups of bacteria, but it has more potential against Gram negative organisms such as *P. aeruginosa* and *E. coli* than the Gram positive, i.e., *S. aureus*. The Ag nanoparticles have shown more antimicrobial activity against Gram negative bacteria than the Gram positive bacteria. This could be because Gram negative bacterial cell wall contains thin layer of peptidoglycan whereas Gram positive bacterial cell wall contains thick layer (Le et al. 2010). The most of the disease causing Gram negative bacteria are transmitted through water, so the synthesized Ag nanoparticles impregnated with the polymer could be used for water purification filter systems (Jain and Pradeep, 2005).

The SEM micrograph of AgNPs treated with *P. aeruginosa* confirms the bactericidal activity of nanoparticles synthesized by *C. utilis*. The detailed mechanism of interaction and penetration of silver nanoparticles inside the cell has not been fully studied. The electrostatic attraction between negatively charged cell membranes and positively charged nanoparticles could be responsible for the interactions between nanoparticles and bacterial cells (Raffi et al (2008). The bactericidal activity of silver nanoparticles against pathogenic bacteria could be used in conjunction with advances in impregnation techniques and polymer technology to expand the range of applications of these nanoparticles in the preservation of food, disinfection of medical supplies and equipment, and decontamination of the surfaces of items such as toys and kitchenware (Matsumura et al. 2003). Besides their bactericidal activity and immediate antibacterial effect against a wide variety of pathogenic bacteria, silver nanoparticles have particular characteristics provided by the silver itself. It tends to induce low bacterial resistance and has low toxicity and minimal side effects after absorption by the body (Ip et al. 2006).

## Conclusion

The present study concludes ecofriendly production of silver nanoparticles using yeast *C. utilis* NCIM 3469. It has more potential activity against Gram negative bacteria. Ag nanoparticles accumulate on the cell surface which causes cell membrane lysis as confirmed by SEM. The antibacterial activity of biofabricated AgNPs revealed applicability in the preparation of reactors for the treatment of waste water and in the development of antibacterial water filters for treatment of water.
